# Multilevel analysis of systolic blood pressure and ACE gene I/D polymorphism in 438 Swedish families – a public health perspective

**DOI:** 10.1186/1471-2350-7-14

**Published:** 2006-03-01

**Authors:** Juan Merlo, Kristina Bengtsson-Boström, Ulf Lindblad, Lennart Råstam, Olle Melander

**Affiliations:** 1 Department of Health and Health Care Management (Epidemiology), Region Skåne, Lund, Sweden.; 2 Community Medicine and Public Health research group, Department of Clinical Sciences, Malmö University Hospital, Faculty of Medicine, Lund University, Malmö, Sweden; 3 Wallenberg Laboratory, Department of Clinical Sciences, Malmö University Hospital, Faculty of Medicine, Lund University, Malmö, Sweden

## Abstract

**Background:**

Individuals belonging to the same family share a number of genetic as well as environmental circumstances that may condition a common SBP level. Among the genetic factors, the angiotensin converting enzyme (ACE) gene I/D polymorphism appears as a possible candidate as it might influence both SBP and the pharmacological effect of ACE inhibitors. We aimed to combine genetic epidemiology with public health ideas concerning life-course and multilevel epidemiology in order to understand the role of familial factors regarding individual SBP.

**Methods:**

We applied multilevel regression analysis on 1926 individuals nested within 438 families from South Sweden. Modelling familial SBP variance as a function of age and use of ACE inhibitors we calculates a variance partition coefficient and the proportional change in familial SBP variance attributable to differences in ACE gene I/D polymorphism

**Results:**

Our results suggest the existence of genetic or environmental circumstances that produce a considerable familial clustering of SBP, especially among individuals using ACE-inhibitors. However, ACE gene I/D polymorphism seems to play a minor role in this context. In addition, familial factors – genetic, environmental or their interaction – shape SBP among non-users of ACE inhibitors but their effect is expressed later in the life-course.

**Conclusion:**

Strategies directed to prevent hypertension should be launched in younger rather than in older ages and both prevention of hypertension and its treatment with ACE inhibitors should be focused on families rather than on individuals.

## Background

Individuals belonging to the same family share a number of genetic as well as environmental factors like common physical environment, socioeconomic position, dietary habits, familial attitudes and health believes, etc. Such circumstances may condition a common level of systolic blood pressure (SBP) that may express itself as a clustering of SBP level within families.

It is known that SBP is a central risk factor for cardiovascular diseases [1]. SBP level increases with age and also is conditioned by genetic factors [2]. Moreover, in presence of established hypertension pharmacological treatment of blood pressure is as well a strong determinant of SBP level for obvious reasons.

One of the main mechanisms for blood pressure homeostasis is the renin angiotensin aldosterone system (RAAS) and a common effective group of antihypertensive drugs are angiotensin converting enzyme (ACE) inhibitors. By blocking the ACE these drugs reduce the levels of angiotensin II and thereby decrease vasoconstriction and renal sodium reabsorption which, in turn, leads to lower blood pressure [3]. Among the genetic factors behind a possible familial clustering of SBP, the angiotensin converting enzyme (ACE) gene I/D polymorphism appears as a possible candidate. ACE levels are highly inherited and show a great interindividual variation [4]. It has been shown that the ACE gene (chromosome 17q23) harbours an insertion/deletion (I/D) polymorphism that accounts for 47% of the variance in plasma ACE concentration [5]. However, it is possible that the I/D polymorphism per se may not be responsible for the ACE levels [6] but rather an unknown nearby polymorphism that is in strong linkage disequilibrium with the ACE gene I/D locus [7]. It is thus plausible that the pharmacological effect of ACE inhibitors drugs could be modified by familial factors, particularly by familial differences in the prevalence of the ACE gene I/D polymorphism. It has also been suggested that ACE gene polymorphism may modulate the physiological age related changes in blood pressure [8,9].

Therefore, we expected to find a familial clustering for both ACE I/D polymorphism and for SBP level. If this were true, it might also be possible that familial differences in ACE I/D polymorphism could explain some of the familial differences in SBP and thus clustering of SBP. This possible phenomenon might differ by age and use of ACE inhibitors.

Considering all those factors (i.e. family environment, blood pressure, individual age, pharmacological treatment with ACE inhibitors and the ACE gene I/D polymorphism) simultaneously is a methodological challenge. In the present study we aim to apply a methodological approach based on multilevel regression analysis [10,11] that may be useful in this context [12,13]. By doing so, we aimed to combine genetic epidemiology with public health ideas concerning life-course and multilevel epidemiology in order to understand the role of familial factors regarding individual SBP.

## Methods

### Participants

In this study we pooled three population based family materials from South Sweden consisting of at least two siblings and, when available, their relatives and spouses. The information was collected during 1996–2000 in all the three samples. Two of them (Malmö-B and Skara) included siblings with established hypertension, whereas one (Malmö A) included siblings with a positive family history of established hypertension but presence of established hypertension was not a necessary inclusion criterion. In Malmö-A, probands were identified in the Malmö Diet and Cancer [14] and Malmö Preventive Project [15] databases. In Malmö B, probands were identified at five different primary health care centres in southern Sweden. In the Skara sample, probands and their families were ascertained from patients diagnosed with hypertension from two earlier population based surveys in Skara, one investigation of patients with hypertension and diabetes in primary health care [16], and one investigation of a random sample of Skara population [17].

The local ethics committee at Gothenburg and Lund University approved the study protocols and all participants gave their informed consent.

### Assessment of blood pressure

All subjects were investigated in the morning after at least 10-hours fast. Blood pressure was measured in the right brachial artery with subjects in the supine position after at least 5–15 minutes rest by specially trained nurses using a using Tricuff^® ^sphygmomanometer for adjustment of cuff size to arm circumference [18]. The systolic and diastolic blood pressures were recorded to the nearest 2 mmHg and the mean of three measurements was recorded. Established hypertension was defined as having at least three consecutive blood pressure recordings at different occasions ≥ 160 mmHg systolic and/or ≥ 90 mmHg diastolic or being on medication for treatment of blood pressure.

### Genotyping

High molecular DNA was extracted from blood leucocytes using standard techniques [19]. The ACE gene I/D polymorphism was genotyped according to the methods described by Rigat et al [20] except that 1.5 mmol MgCl_2 _and 1% formamid were used in the PCR reaction. To exclude mistyping of the heterozygotes as DD homozygotes all the DD genotypes samples were confirmed with an insertion specific PCR [21].

### Use of medication

In order to assess current pharmacological treatment the subjects were asked to bring receipts and pill boxes and the information was asserted by a face to face interview performed by specially trained nurses. ACE inhibitors were defined according the Anatomical Therapeutic Chemical classification system code C09A [22].

### Statistical analysis: multilevel regression analysis

Individual SBP was analyzed by multilevel linear regressions models [10,23] with individuals at the first level and families (i.e., pedigrees) at the second level. Multilevel regression analysis is a very appropriate technique for performing variance analysis in unbalanced clusters as families [23].

We fitted three models. *Model I *did not include any explanatory variables and only focused on decomposing the total SBP variance (V_Total_) in its individual (V_I_) and familial (V_F_) components. In *model II *we included dummy variables for the original study samples (Skara, Malmö-A and B) individual age, and for use of ACE inhibitors. In the last *model III *we included the ACE gene I/D polymorphism in addition to the variables already included in *model II*. For the statistical analysis we constructed dummy variables for the presence or absence of homozygosis II, homozygosis DD, and heterozygosis ID, and used homozygosis II as reference in the comparisons. In model II and III we investigate family-individual interaction by allowing the slope of the association between on the one hand SBP and on the other age or ACE inhibitors to be random at the family level. That is, we investigate whether the family level modified those individual level associations.

We calculated the Variance Partition Coefficient (VPC). The VPC is a measure of the extent to which members of a family resemble each other more than they resemble individuals from other families in relation to SBP level. The VPC is the percentage of the total variance (V_F _+ V_I_) in SBP that is attributed to the family level (V_F_) and is, therefore, a measure of *clustering*. In the simplest form (model i) the VPC corresponds to the intraclass correlation coefficient, being a measure of general clustering of individual SBP in the families.

VPC=VFVF+VI×100
 MathType@MTEF@5@5@+=feaafiart1ev1aaatCvAUfKttLearuWrP9MDH5MBPbIqV92AaeXatLxBI9gBaebbnrfifHhDYfgasaacH8akY=wiFfYdH8Gipec8Eeeu0xXdbba9frFj0=OqFfea0dXdd9vqai=hGuQ8kuc9pgc9s8qqaq=dirpe0xb9q8qiLsFr0=vr0=vr0dc8meaabaqaciaacaGaaeqabaqabeGadaaakeaacqqGwbGvcqqGqbaucqqGdbWqcqGH9aqpdaWcaaqaaiabbAfawnaaBaaaleaacqqGgbGraeqaaaGcbaGaeeOvay1aaSbaaSqaaiabbAeagbqabaGccqGHRaWkcqqGwbGvdaWgaaWcbaGaeeysaKeabeaaaaGccqGHxdaTcqaIXaqmcqaIWaamcqaIWaamaaa@3E68@

In models II and III, because of the existence of random slopes at the familial level the variance of the slope of the association between SBP and age as well as the variance of the slope of the association between SBP and use of ACE inhibitors are also a coefficient in a function that describes how the between-families variation in SBP differs in individuals with different age and use of ACE inhibitors. This variance function includes also the intercept variance and the covariances. The output of this variance function was obtained from the software used for the multilevel regression analysis. [11,24] By means of this variance function we calculated the VPC as a function of age and use of ACE inhibitors which provided information on the relevance of familial factors for understanding SBP differences in specific groups of individuals (i.e., defined by age and use of ACE inhibitors). A large VPC would indicate that familial factors are important in order to understand individual SBP differences. On the other hand, a VPC close to zero would indicate that the families exert only a small influence on individual SBP variance.

Using the V_F _from the models before (Model II) and after (Model III) adjustment for ACE I/D gene polymorphism we calculated the absolute [V_F _before - V_F _after] and the relative [((V_F _before - V_F _after)/V_F _before) × 100] change in variance.

The precision of the estimates was appraised by their SE. Parameters were tested in an approximate manner using a normal test (i.e., the ratio of an estimated variance to its standard error) and Wald statistic with a chi-square distribution [25], and calculated exact *p*-values. A *p*-value greater than 0.05 was considered non-significant (NS).

In addition to familial clustering (i.e., VPC) of SBP we also calculated the familial clustering of homozygosis II, homozygosis DD, and heterozygosis ID, as well as the familial clustering of ACE inhibitors use. Since these variables are binary we applied multilevel logistic regression analysis [25] and calculated the VPC using the latent variable threshold method recommended by Snijders [23].

The software MLwiN, Version 2 [24] was used to perform the analyses. Regression parameters were estimated using Markov Chain Monte Carlo (MCMC). We use the Deviance Information Criterion (DIC) to compare the goodness of the fit of consecutive models. [24]

## Results

The characteristics of the population are presented in Table 1. Mean SBP was lowest in Malmö-A and highest in Skara. The prevalence of the different forms of ACE gene I/D polymorphism showed slight differences between the three pooled datasets (p = 0.014), and the prevalence of use of ACE inhibitors was highest in Skara and lowest in Malmö-A. Table 2 shows the distribution of the families according their size. The number of relatives varied but most of the families had between two and five relatives.

**Table 1:** Characteristics of the 1926 individuals from 438 Swedish families with history of hypertension

	Whole Sample	Malmö Sample A	Sample B	Skara
		
Number of people	1926	581	1100	245
Number of families	438	145	223	70
Age (mean years)	54	51	54	60
Men (%)	47	49	47	41
Systolic blood pressure (mean mmHg)	139	131	142	146
Use of ACE-inhibitors (%)	10	8	11	13
ACE gene I/D polymorphism				
II (%)	23	23	23	22
ID (%)	48	52	47	48
DD (%)	26	24	29	21

ACE = Angiotensin converting enzyme inhibitors. I = insertion, D = deletion

**Table 2:** Family size in the study sample

Family size	Number of individuals	Number of families	Percentage
2	120	60	13.7
3	321	107	24.4
4	512	128	29.2
5	260	52	11.9
6	192	32	7.3
7	133	19	4.3
8	112	14	3.2
9	108	12	2.7
10	60	6	1.4
≥11	108	8	1.8
Total	1926	438	100

Table 3 shows the results of the multilevel linear regression analysis. The regression coefficients in the fixed effects section of the model II informs that for each year of age SBP increases by 0.9 mmHg. Users of ACE inhibitors have in average 4.5 mmHg higher SBP than non-users (i.e., users of other antihypertensive medication or individuals without pharmacological treatment of hypertension). Inclusion of the ACE gene I/D polymorphism in model III did not affect the associations (regression coefficients) estimated in model II. Compared with individuals homozygous for II, heterozygous ID and homozygous DD individuals presented a lower SBP but this difference was only significant for the ID group (p = 0.025).

**Table 3:** Multilevel regression analysis of systolic blood pressure (SBP) in 1926 individuals from 438 Swedish families with hypertension

	**Model I (empty)**	**Model II**	**Model III**
	
**Fixed effects**			
	*Intercept (mmHg)*		
	139.17 (0.64)	141.78 (1.50)	143.23 (1.66)
	*Regression coefficient (SE)*		
	
Age (years)	-	0.90 (0.03)	0.90 (0.03)
Sex (men vs. women)	-	-0.23 (0.81)^NS^	-0.20 (0.83)^NS^
ACE inhibitors use (yes vs. no) Cohort	-	4.51 (1.66)	4.33 (1.62)
Skara	-	Reference	Reference
Malmö A	-	-7.64 (1.68)	-7.49 (1.67)
Malmö B	-	-0.96 (1.60)^NS^	-0.87 (1.59)^NS^
ACE gene I/D polymorphism			
II	-	-	Reference
ID	-	-	-2.38 (1.06)
DD	-	-	-1.48 (1.21)^NS^
**Random effects**			
	*Variance (SE)*		
	
Individual level	511.50 (19.00)	290.01 (11.17)	290.32 (11.02)
Familial intercept	50.40 (12.34)	30.22 (6.45)	28.58 (7.56)
Age-SBP slope	-	0.04 (0.01)	0.04 (0.01)
ACE inhibitors-SBP slope	-	142.46 (54.90)	131.04 (48.83)
	*Covariance (SE)*		
	
Age-intercept	-	0.99 (0.21)	0.96 (0.23)
ACE inhibitors-intercept	-	-22.62 (16.51)	-18.40 (14.62)
Age-ACE inhibitors	-	-0.70 (0.61)	-0.66 (0.59)
	*VPC (%)*		
	
	9	See figure 2	See figure 2

**Goddness of the fit**			
DIC (MCM)	16978	15898	15897

VPC: Variance partition coefficient. DIC: Deviance Information Criterion. MCMC: Markov Chain Monte Carlo

**Figure 1:**
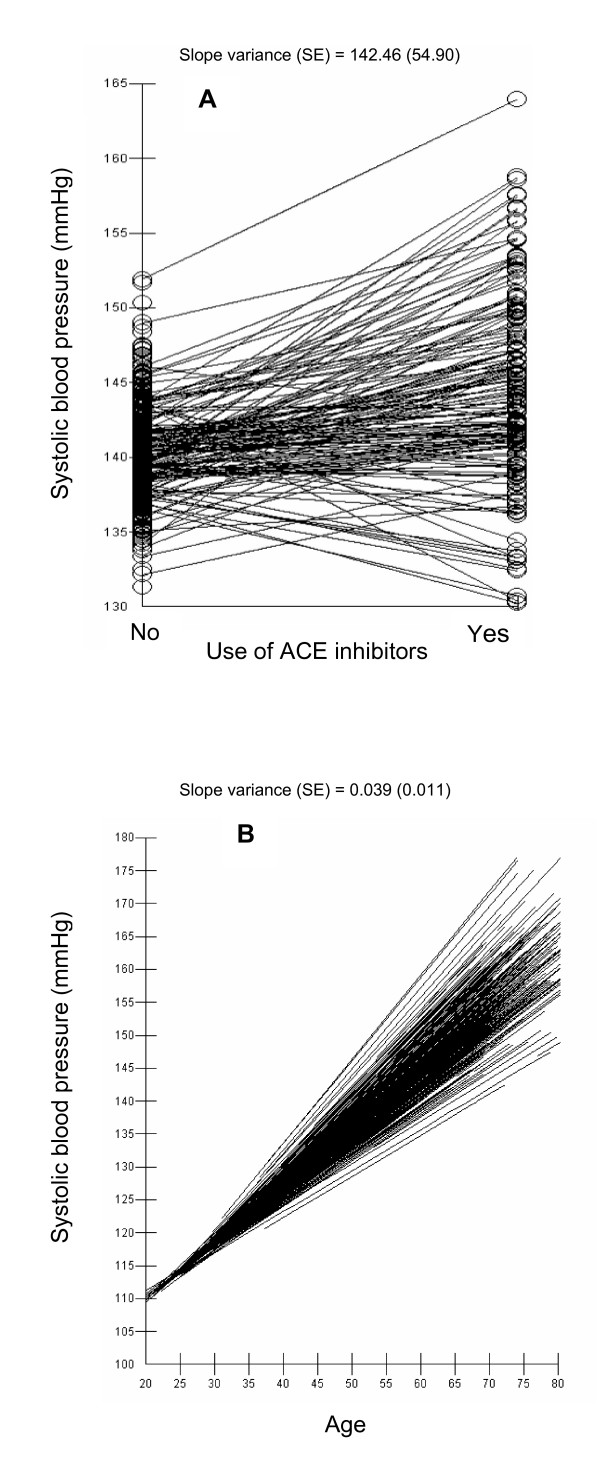
shows the differences in the slope (each line represents a family) of the association between on the one hand age (A) and use of ACE inhibitors (B) and other hand SBP level. Simple visual observation suggests that there is considerable familial heterogeneity (i.e., family-individual interactions) in those associations. Observe that the regression lines are model predictions obtained in model II.

Table 3 also presents the components of variance analysis (i.e. random effects). Model I informed that individuals from the same family shared a common level of SBP since 9% of the individual differences in SBP were actually at the family level (VPC = 9%). Inclusion of random slopes for age and use of ACE inhibitors in model II discovered a considerably familial heterogeneity. Figure 1 shows the familial differences in the slope of the association between on the one hand age (A) and use of ACE inhibitors (B) and on other hand SBP level. Simple visual observation suggests that familial differences in SBP increased by age and were higher in users of ACE inhibitors.

**Figure 2:**
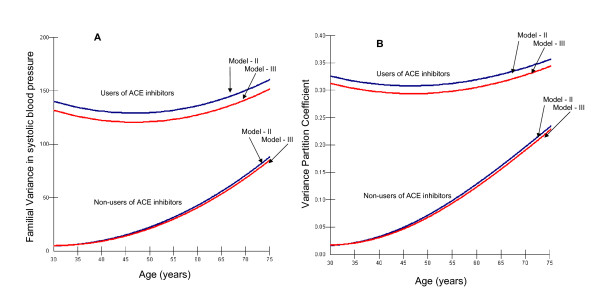
illustrates the variance between families in SBP and the familial clustering of SBP level (i.e., VPC) along individual age and in users and non-users of ACE-inhibitors. Because of the existence of slope variance (Figure 1) the familial SBP variance (Figure 2A) and the VPC (Figure 2B) became a function of age and use of ACE inhibitors. Familial clustering of SBP (i.e., VPC) seems increase with age and this effect is more evident among non users of ACE inhibitors. In users of ACE inhibitors familial clustering of SBP is already evident in younger ages. Inclusion of the ACE gene I/D polymorphism in model III (red colour) reduces the familial clustering of SBP even if this effect is small. Consider that the estimations are insecure at ends of the curves since the number of individuals is small. The information illustrated in figure 2 is also numerically presented in table 4.

Figure 2 illustrates that because of the existence of slope variance the familial SBP variance (Figure 2A) and the VPC (Figure 2B) became a function of age and use of ACE inhibitors. In users of ACE inhibitors familial variance and clustering of SBP are already evident in younger ages, but in non-users of ACE inhibitors these parameters seem increase with age. Inclusion of the ACE gene I/D polymorphism in model III (red colour) reduces slightly the familial clustering of SBP among users of ACE inhibitors but it did not improve the statistical goodness of the fit (Table 3). The information presented in figure 2 is also numerically illustrated in table 4.

The results of the complementary logistic regression analyses showed a strong familial genetic clustering for the different forms of the ACE gene I/D polymorphism, specially for homozygous forms DD (VPC = 43%) and II (VPC = 37%) and in a lower degree for the heterozygous form ID (VPC = 15%). Use if ACE inhibitors was also clustered within families (VPC = 9%).

## Discussion

The multilevel regression analysis allowed us to study the distribution of blood pressure in families along individual age and use of ACE inhibitors and simultaneously investigate the role of ACE gene I/D polymorphism in this context.

We observed that SBP level clustered within families to an appreciable degree, and that this clustering increased considerably with age in people that did not use ACE-inhibitors. This fact suggests the existence of specific familial genetic or environmental factors influencing individual SBP, and that the effect of these familial factors express later in the life course. In fact, familial differences in SBP were large in the elderly but very small in youth people.

It is possible that both familial genetic and environmental circumstances (e.g., common physical environment, socioeconomic position, dietary habits, familial attitudes and health believes, etc) affecting SBP act only after latency period. It is also probable that genetic susceptibility affects SBP level only when the effects of familial environmental factors become fully developed through the individual's life course. As a hypothetical example, learned familial behaviours may lead to obesity in adulthood, which in turn increases the risk of hypertension later in life in people with certain genetic predisposition. It is possible that these kinds of mechanisms could explain our results. However, the database used in this investigation was not primarily design to answer those question and information on socioeconomic and behavioural factors was not available. Moreover our study was cross-sectional and we cannot exclude – even if we dot believe it probable – the existence of a cohort effect confounding the age related familial differences in SBP.

In the present study we cannot differentiate between genetic effects (apart from the minor effect of the ACE I/D polymorphism) and effects of familial environment on SBP. However, it is reasonable to believe that, to a large extent, genetic and environmental factors determining SBP interact with each other throughout life. Thus, genetic susceptibility to high SBP is likely to be modified by reducing environmental risk factor load. Therefore, our results suggest that acting on familial environmental factors early in the life course, rather than in late adulthood, might be a more effective strategy to prevent high blood pressure in the population. [26] Future research will identify which those factors and how we can influence them.

The degree of familial clustering of SBP level was much higher in users of ACE-inhibitors than in non-users (Figure 2B) and this clustering was also considerably less age dependent. This observation indicates that ACE inhibitors produce a more intense blood pressure lowering effect in some families than in others. One reason for this familial heterogeneity might be genetic differences in the susceptibility to the effect of ACE inhibitor, and our hypothesis was that the ACE I/D polymorphism could be a candidate for explaining these familial differences. We also observed that – as expected – the different forms of the ACE I/D polymorphism presented a high degree of clustering within families. However, accounting for differences in the ACE I/D polymorphism explained only a minor part of the familial differences in SBP level among user of ACE inhibitors.

The role of the ACE I/D polymorphism in blood pressure regulation has been extensively studied. Although there is no doubt that the polymorphism is associated with plasma ACE activity, there is less evidence on the role of ACE I/D polymorphism in blood pressure regulation. [5,27] Blood pressure is a complex trait that results from the effect of multiple genes and many environmental influences [28]. There is a growing agreement from many studies in that single gene effects on antihypertensive drug responses are small, and even the combined effects of all presently known polymorphisms do not account for enough variation in response to be clinically useful.[29] With this in mind, it is not surprising that the ACE I/D polymorphism only explained a very small part of the total familial variance in SBP level. Nevertheless our results suggest that the ACE I/D polymorphism might have some role in blood pressure control among users of ACE inhibitors.

ACE-inhibition was not a first-line antihypertensive therapy in Sweden at the time of patient-recruitment in the present study, indicating that these patients had responded less well on conventional treatment (thiazides and beta-blockers) or that they had concomitant diseases such as diabetes with microalbuminuria or heart failure. One possible explanation for the greater familial clustering of SBP in users than in non-users of ACE inhibitors could thus be that patients treated with ACE-inhibitors had more atherosclerosis and endothelial dysfunction than non-users of ACE inhibitors. In analogy with the effect of age on familial SBP clustering in non-users, it can be speculated that genetic and familial environmental factors affecting SBP have a greater impact when acting on the background of a relatively more pathological or "biologically aged" vascular system.

Most patients treated with ACE inhibitors were also on treatment with other antihypertensive agents, mostly beta-receptor blockers (i.e., 32%) and calcium channel blockers (i.e., 27%) introducing some uncertainty as to how much of the on-treatment blood pressure level was influenced by overall drug effects as opposed to ACE-inhibitor specific effects. However, adjustment for use of those medications in model III had only a minor effect on the slope variance, which was reduced from 131 to 128 (not show in tables).

## Conclusion

We advocate a study design that may be useful for studying the influence of genetic and environmental familial factors in public health. This approach may facilitate the investigation of common processes that with multifaceted expression and development across time in different contexts. Multilevel regression analysis [10] emerges as an appropriate tool for investigating complex patterns of variance, an aspect that is especially useful in genetic epidemiology where the information has a natural hierarchical structure (e.g., pedigrees, generations, individuals) [12,13].

Our study suggest that familial circumstances (genetic, environmental or their interaction) condition individual SBP level and seems to modify the effect of ACE inhibitors, a fact that deserves more investigation in order to improve the effectiveness of pharmacological treatment of hypertension. Familial circumstances also seem to play a relevant role for understanding SBP level in non-users of ACE inhibitors but those effects express later in the life course. Those facts suggests that strategies directed to prevent hypertension should be launched in younger rather than in older ages and both prevention of hypertension and its treatment with ACE inhibitors should be focused on families rather than on individuals.

## Competing interests

The author(s) declare that they have no competing interests.

## Authors' contributions

JM had the original idea of this article, performed the design, the analyses and the discussion of the results and write the manuscript. OM and KB contributed to the design, the discussion of the results and the writing of the manuscript. UL and LR contributed to the discussion of the results and the writing of the manuscript. OM, KB, UL and LR supplied the databases. All authors read and approved the final manuscript.

## Pre-publication history

The pre-publication history for this paper can be accessed here:


